# Influence of musculotendon geometry variability in muscle forces and hip bone-on-bone forces during walking

**DOI:** 10.1371/journal.pone.0222491

**Published:** 2019-09-25

**Authors:** E. Martín-Sosa, J. Martínez-Reina, J. Mayo, J. Ojeda

**Affiliations:** Departamento de Ingeniería Mecánica y Fabricación, Universidad de Sevilla, Seville, Spain; Virginia Tech, UNITED STATES

## Abstract

Inverse dynamics problems are usually solved in the analysis of human gait to obtain reaction forces and moments at the joints. However, these actions are not the actual forces and moments supported by the joint structure, because they do not consider the forces of the muscles acting across the joint. Therefore, to analyse bone-on bone forces it is necessary to estimate those muscle forces. Usually, this problem is addressed by means of optimization algorithms. One of the parameters required to solve this problem is the musculotendon geometry. These data are usually taken from cadavers or MRI data from several subjects, different from the analysed subject. Then, the model is scaled to the subject morphology. This procedure constitutes a source of error. The goals of this work were two. First, to perform a sensitivity analysis of the influence of muscle insertion locations on the muscle forces acting on the hip joint and on the hip joint bone-on-bone forces. Second, to compare the hip joint bone-on-bone forces during gait cycle obtained through muscle insertion locations taken from a musculoskeletal model template and a scaling procedure to those obtained from a subject-specific model using an MRI of the subject. The problem was solved using OpenSim. Results showed that anatomical variability should be analysed from two perspectives. One the one hand, throughout the gait cycle, in a global way. On the other hand, at a characteristic instant of the gait cycle. Variations of ±1 cm in the position of the attachment points of certain muscles caused variations of up to 14.21% in averaged deviation of the muscle forces and 58.96% in the peak force in the modified muscle and variations up to 57.23% in the averaged deviation of the muscle force and up to 117.23% in the peak force in the rest of muscles. Then, the influence of that variability on muscle activity patterns and hip bone-on-bone forces could be described more precisely. A biomechanical analysis of a subject-specific musculoskeletal model was carried out. Using MRI data, variations up to 5 cm in the location of the insertion points were introduced. These modifications showed significant differences between the baseline model and the customized model: within the range [-12%, 10%] for muscle forces and around 35% of body weight for hip bone-on-bone forces.

## 1. Introduction

Movement analysis techniques are useful tools to study human locomotion. Two of the most interesting aspects are the estimation of muscle forces carried out during movement and loads supported by joints, the so-called bone-on-bone forces. The latter is a widely studied problem in Biomechanics but continues being a topic of interest due to the importance of a correct estimation of the loads applied to the joints in clinical studies or in sport sciences [1–8], among others. Regarding clinical applications, the estimation of bone-on-bone forces is useful in rehabilitation procedures [2,8] or in prosthesis design problems [3]. In the sport sciences area, the information about the loads supported by joints is crucial to prevent injuries and to optimize the execution of a certain task [7].

One of the most usual approaches employed in the estimation of bone-on-bone forces is solving an inverse dynamics problem [9–11]. The solution of this problem yields joint forces and moments that balance the external forces: gravitational, inertial or externally applied forces. However, those actions are not the real forces and moments supported by the joint structure, because they do not take into account the muscle forces acting on the joint [11]. To analyse the actual bone-on-bone forces it is necessary to estimate muscle forces previously. Usually, this problem is addressed by means of optimization algorithms [12–13].

The estimation of muscle forces and bone-on-bone forces is usually made *in silico* and mainly with the use of Multibody Techniques to simulate the musculoskeletal model. One software that implements these techniques is OpenSim [14], an open source software which is extensively used nowadays. This software has a library of model templates which need to be scaled to the subject’s morphology before running simulations. Therefore, these procedures do not consider the actual morphology of the subject, but they estimate it from a database. This is a source of error whose effect needs to be investigated. Thus, sensitivity analysis can be a powerful tool to assess the influence of scaling on outputs of musculoskeletal models.

Many sensitivity analyses dealing with joint reactions or muscle forces can be found in the literature. However, the number of those works addressing the problem from a mechanical point of view is quite smaller. In fact, most of the works that can be found in the literature are focused on bone-on-bone forces [15–18], and just a few are focused on muscle forces [19–20]. Among the former, some works have studied the influence of modifying muscle properties [15–17]. Wesseling et al [15] and Ardestani et al [16], modified the strength parameters of musculoskeletal models. Saliba et al [17] quantified the sensitivity of knee bone-on-bone forces to simultaneous errors in frontal plane knee alignment and contact locations.

Some studies published in the literature have carried out sensitivity analyses to study the effect of modifying the origin and insertion points of muscles on the exerted forces [18–20]. More precisely, Carbone et al [19], modified the attachment points of certain muscles, but these authors modified some muscle groups as a single actuator and ignored the effect of each muscle separately. The variable defined to analyse the effect of anatomical variability was the deviation of muscle forces from those obtained with a nominal (or baseline) model, averaged throughout the gait cycle. Bosmans et al [20] also studied the influence of the location of attachment points on muscle forces. This study analysed all muscles crossing the right hip, whose insertion points were modified in a representative anatomical quantity. However, these authors only studied the influence that those changes had on a single instant of the gait cycle, t_nom_, the instant at which muscle force reaches its peak, and not throughout the whole gait cycle.

None of the previously cited studies [19–20] analysed the effect that the change of the location of attachment points has on bone-on-bone forces. A sensitivity analysis of these forces was carried out by Valente et al [18]. This study analysed bone-on-bone forces in the hip, knee and ankle, but modified a smaller number of muscles than [19–20]. In addition, this study was not focused on evaluating the influence of the location of attachment points on bone-on-bone forces separately, since other parameters were modified at the same time. In this sense, it would be interesting to analyse this effect independently.

The main aim of this work was to perform a sensitivity analysis to assess the influence of modifying the origin and insertion points of muscles wrapping the right hip joint on muscle forces and bone-on-bone forces throughout the gait cycle. The sensitivity analysis was carried out by defining two output parameters to evaluate the sensitivity to perturbations of geometry of each muscle force evaluated at a reference instant or averaged throughout the whole cycle. The proposed procedure provided a more complete picture of force patterns. As a particular application of the sensitivity analysis, the location of muscle insertion points was modified using MRI data taken from a certain individual. This example showed the importance of using accurate data in subject-specific models.

## 2. Methods

### 2.1. Musculoskeletal modelling

A lower limb generic musculoskeletal model taken from OpenSim [14] (the so-called “Gait 2392”) was used to carry out the analysis. This model has 23 degrees of freedom and 92 actuators. The present study was focused on the right leg, which was made up of 4 bodies: femur, tibia, foot and pelvis. The hip was modelled as a ball-and-socket joint. The knee was modelled as a custom joint where the three relative rotations and the antero-posterior relative displacement were defined as a function of the knee flexion-extension movement. Finally, the ankle was considered as a revolute joint. The patella was not considered and thus the attachment of those muscles inserted in it (only the rectus femoris among the muscles considered in the present study) had to be modelled using moving-points.

All muscles crossing the right hip joint were considered in the analysis (Table 1). The number of muscles was n = 19 in the baseline model and they were modelled as lineal actuators with two attachment points: origin and insertion. Those muscles which are very long or with a complex shape were modelled with auxiliary or intermediate points. These points were called: pseudo-origin (the most distal intermediate point on the proximal segment), pseudo insertion (the most proximal intermediate point on the distal segment) and via-point (intermediate points), as in [19–20].

**Table 1:** Studied muscles.

Muscle	Hip function					
	Abd	Add	Flex	Ext	I rot	E rot
Gluteus maximus anterior/middle/posterior	x	x		x		
Gluteus medius anterior/middle/posterior	x		x	x	x	x
Gluteus minimus anterior/middle/posterior	x	x	x	x	x	x
Perineus	x					x
Sartorius	x		x			
Tensor fasciae latae	x		x		x	
Adductor brevis		x	x			
Adductor longus		x	x	x		
Adductor magnus anterior/middle/posterior		x		x		
Gracilis		x	x			
Iliacus			x		x	
Pectineus		x	x			
Psoas			x		x	
Rectus femoris			x			
Gemellus						x
Biceps femoris long head		x		x		
Quadriceps femoris						x
Semimembranosus		x		x		
Semitendinosus		x		x		

Set of muscles analysed in the study and their functions.

Some muscles, gluteus medius, minimus and maximus and the adductor magnus, were modelled as a combination of three actuators. The names of these actuators were: anterior, middle and posterior. (Table 1).

**Fig 1:**
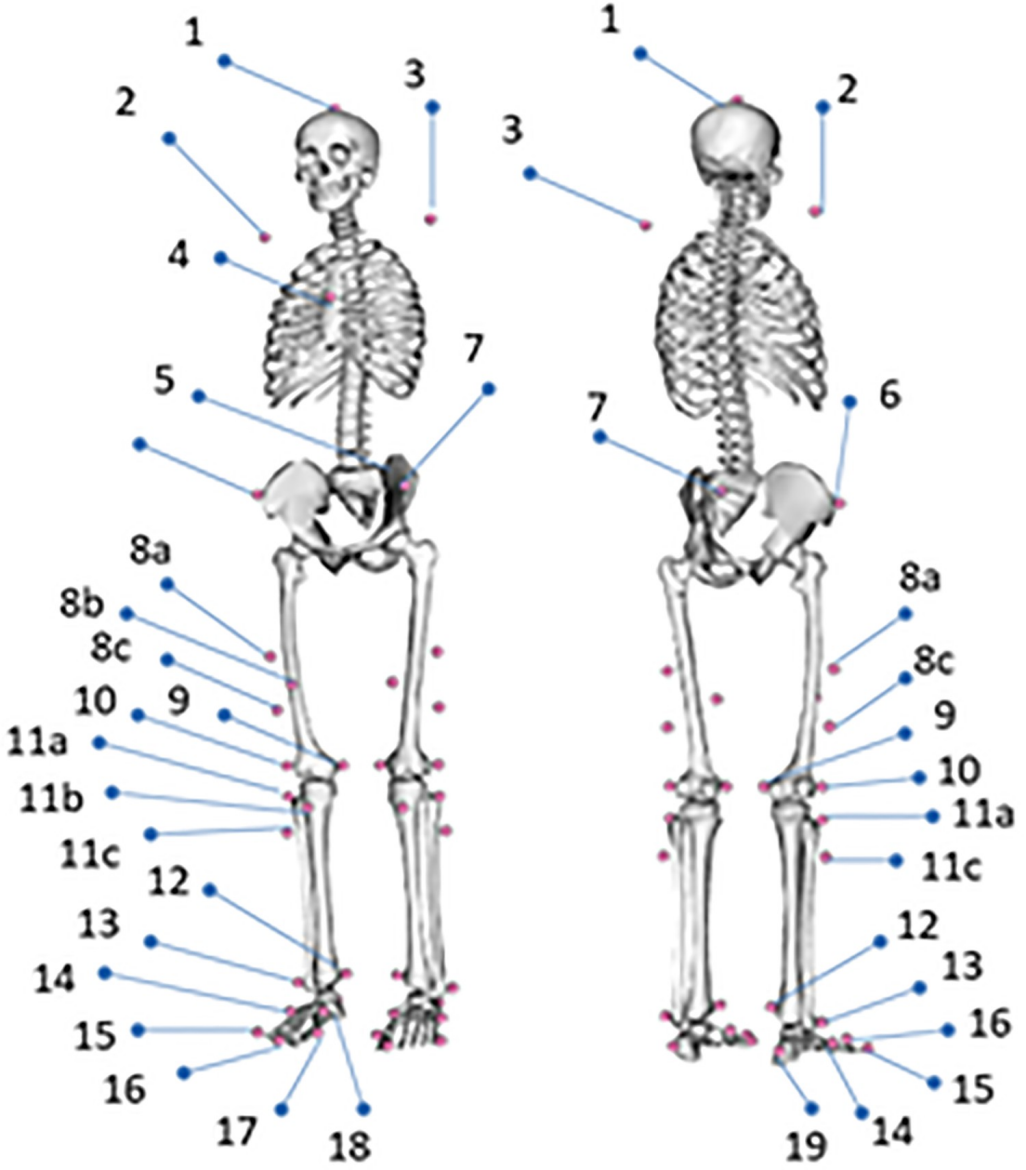
Marker placement protocol.

Gait analysis was simulated for one adult. A male 28 years old person with no bone and gait pathologies was selected for this study. The subject signed an informed consent for its participation in the study and the study protocol was approved by a medical ethics committee through the Andalusian Biomedical Research Ethics Platform (approval number 20151012181252). A modified Cleveland marker placement protocol [21] was used to define the position and orientation of the different parts of the body (Fig 1 and Table 2). Markers trajectories were measured at 100 Hz using a set of 12 Vicon cameras motion capture system. Ground reactions forces were recorded with 2 AMTI force plates using a sample frequency of 1000 Hz.

**Table 2:** Markers protocol labels.

N° Marker	Label	w	N° Marker	Label	w
1	Top_Head	0.1	11.a	R_Shank_Upper	1
2	R_Acromium	0.5	11.b	R_Shank_Front	1
3	L_Acromium	0.5	11.c	R_Shank_Rear	1
4	Sternum	1	12	R_Ankle_Med	-
5	V_Sacral	10	13	R_Ankle_Lat	-
6	R_ASIS	10	14	R_Midfoot_Lat	1
7	L_ASIS	10	15	R_Midfoot_Sup	1
8.a	R_Thigh_Upper	1	16	R_Toe_Tip	10
8.b	R_Thigh_Front	1	17	R_Toe_Med	1
8.c	R_Thigh_Rear	1	18	R_Toe_Lat	1
9	R_Knee_Med	-	19	R_Heel	10
10	R_Knee_Lat	-			

The markers shown in the table are relative to the right leg. This marker placement protocol uses 39 markers. W: weights assigned to each marker in the inverse kinematics problem. Markers 9, 10, 12, 13 and their equivalent in the left leg only contributed in the scaling procedure.

### 2.2. Simulations of the gait cycle

The first step to simulate the gait cycle was to scale the chosen model to match the anthropometry of a particular subject on whom some markers were placed in given anatomical locations. The positions of these markers were adjusted to build a model of the subject under study, following the scaling protocol implemented in OpenSim. The result of scaling the model to the subject’s anthropometry was defined as baseline model. In this model, muscle attachment points were placed where OpenSim locate them by default, using numerical approximation [22] of cadavers’ data [23–24].

Next, the inverse kinematics problem was solved. The input data of this problem are the experimental trajectories of markers, *x*^*exp*^, and the output is the temporal evolution of the generalized coordinates, *q*. The problem was solved using optimization techniques. In particular, minimizing the following objective function:

$${\text{min}}_{q}\left[\sum_{i\in markers}w_{i}{\left\|x_{i}^{exp}-x_{i}(q)\right\|}^{2}\right]$$ (1)

where *x*_*i*_(*q*) is the position of the virtual marker *i*, which depends on the coordinates values, and *w*_*i*_ is a marker weight [25]. The values of the weights tried to minimize the importance of those markers placed on wobbling masses, such as thigh markers, whereas the weights associated to markers located in anatomical landmarks, like heel markers, had a higher value. Numerical values of *w*_*i*_ are shown in Table 2. The results of the inverse kinematics problem were used as input to solve the inverse dynamics problem by means of the classical equations of motion:

$$M(q)\ddot{q}+C(q,\dot{q})+G(q)=\tau$$ (2)

where *q*, $\dot{q}$ and $\ddot{q}$ are the vectors of generalized positions, velocities and accelerations, respectively; *M* is the mass matrix; *C* is the vector of quadratic velocities; *G* is the vector of external forces including gravitational forces and ground reaction forces and *τ* is the vector of generalized forces regarding motor tasks.

Muscle forces (*F*_*mus*_) were estimated solving the following optimization problem:

$$\begin{array}{cc} & \text{min}J=\sum_{m=1}^{n}{\left(a_{m}\right)}^{p}\\ s.t. & \sum_{m=1}^{n}\left[a_{m}.f\left(F_{m}^{0},l_{m},v_{m}\right)\right]r_{m,j}=\tau_{j} \end{array}$$ (3)

where *n* is the number of muscles in the model; *a*_*m*_ is the activation level of muscle *m* at a discrete time step; $F_{m}^{0}$ its maximum isometric force; *l*_*m*_ its length; *v*_*m*_ its shortening velocity; $f(F_{m}^{0},l_{m},v_{m})$ its force-length-velocity relation; *r*_*m*,*j*_ its moment arm about the *j*^*th*^ joint axis and *τ*_*j*_ is the generalized force acting about the *j*^*th*^ joint axis. The cost function *J* of Eq (3) minimizes the sum of muscle activation squared (*p* = 2) as in [12].

**Fig 2:**
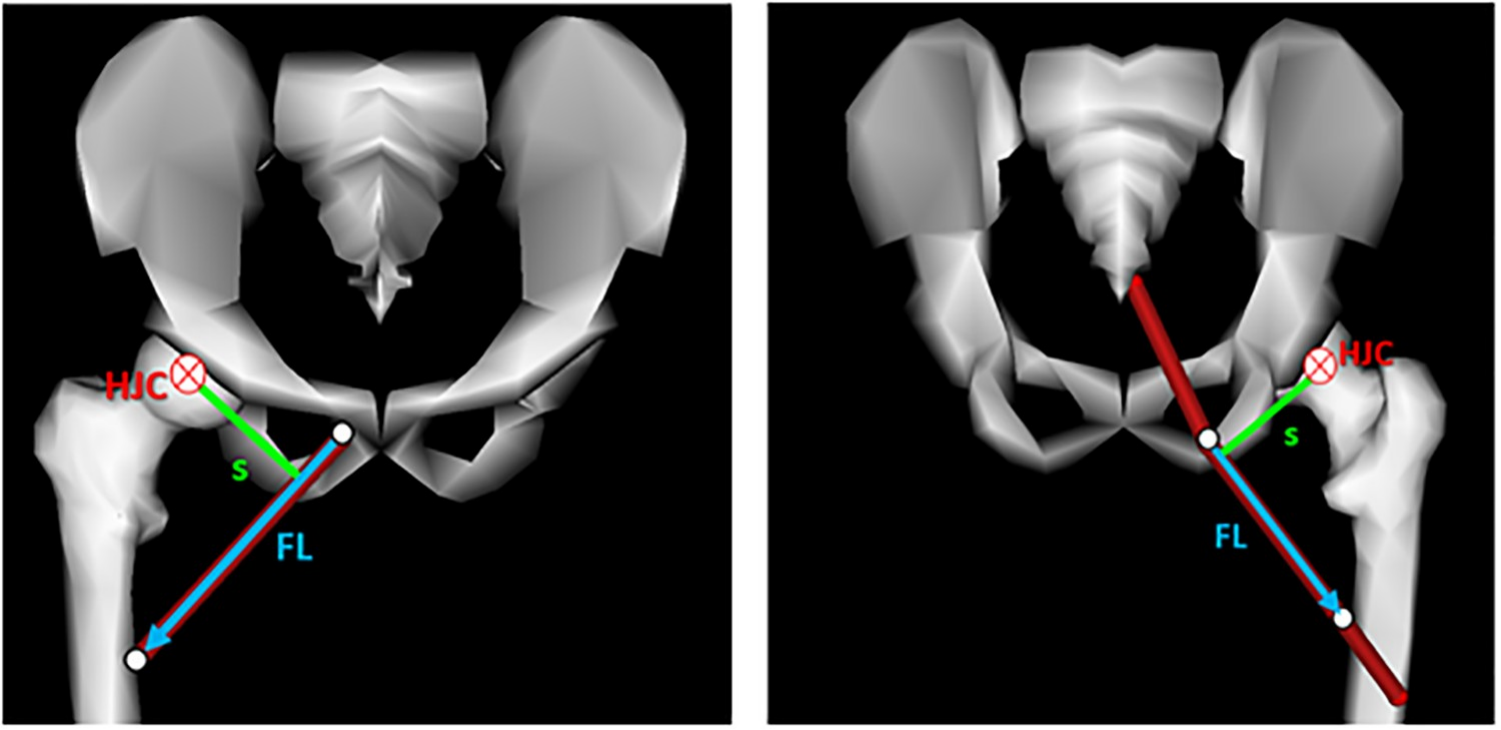
Moment arm definition.

Regarding the moment arms, they are calculated as internal variables in OpenSim and, therefore, the software does not provide them as output time dependent variables. However, due to their importance in the analysis of the present results, they were obtained using a script programmed in Matlab^®^ by means of its formal definition shown in Eq (4): the moment arm was defined as in this type of problems: the cross product of two vectors: the distance, *s*, between the line of action of the muscle force and the position of the joint of interest and the unit vector of the muscle force direction, *FL* (Fig 2, left). In case of muscles with a more complex path, the muscle force direction, *FL*, was defined by two via points belonging to different solids (Fig 2, right).

$$r=\frac{\tau }{\left|F_{mus}\right|}=\frac{s\times F_{mus}}{\left|F_{mus}\right|}=s\times FL$$ (4)

The last step was to calculate bone-on-bone forces. These forces were not the solution of the inverse dynamics problem because that solution does not consider the forces of muscles acting across the joint. Besides, the solution of the inverse dynamics problem was obtained in terms of vector q, the vector of generalized coordinates, which were expressed in a local frame for each segment. These coordinates were transformed into a global Cartesian frame y, to obtain bone-on-bone force components. Then, the vector of reaction forces together with muscle forces were used as input to obtain the joint bone-on-bone forces using a recursive procedure [26] with the following equation:

$$R_{i}=M_{i}(y){\ddot{y}}_{i}-(\sum F_{muscles}+\sum F_{external}+R_{i-1})$$ (5)

In this equation, vector *R*_*i*_ contains the resultant forces and moments at joint *i* or bone-on-bone forces. The body distal to joint *i*, *B*_*i*_, is treated as an independent body with known kinematics in a global reference frame. Thus, *ÿ*_*i*_ represents the six dimensional vector of known angular and linear accelerations of *B*_*i*_, and *M*_*i*_(*q*) is the 6x6 mass matrix of *B*_*i*_; *F*_*external*_ and *F*_*muscles*_ represent the previously calculated forces and moments produced by external loads and musculotendon actuators, respectively. *R*_*i*−1_ represents the joint reaction load applied at the distal joint and it is known since it was calculated in the previous recursive step. Our aim was to study bone-on-bone forces only at the hip joint, thus, the generic name *R*_*i*_ will be replaced from now on by HJF (acronym for hip joint force) and expressed in the local frame attached to the femur. All the simulations were run in OpenSim with the exception of the moment arm calculation.

### 2.3. Anatomical variability

One of the goals of this work was to analyse the influence of the anatomical variability on the estimation of muscle forces and HJF. Therefore, starting from the results provided by the inverse kinematic and kinetic problems, modifications in the location of muscle attachment points were carried out and then, muscle forces and HJF were calculated.

**Fig 3:**
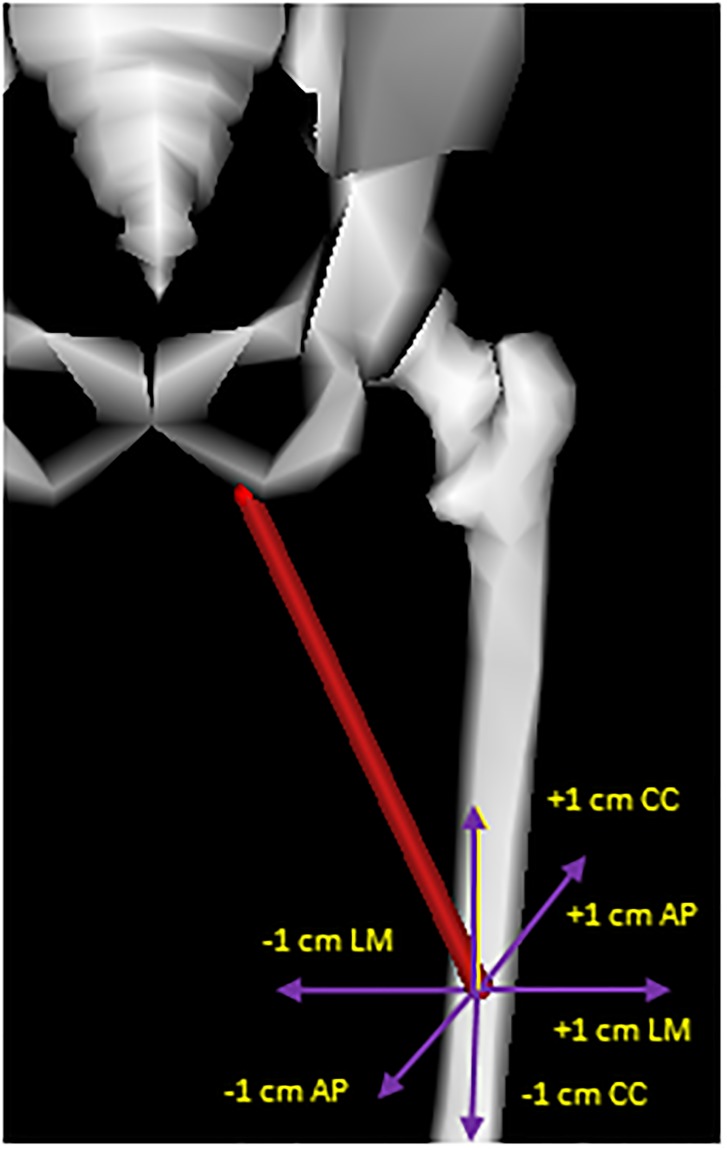
Modification of the location of the insertion point of the adductor magnus middle from the baseline model.

A value of ±1 cm was established in the literature as the reference value to modify attachment points in each direction [20]. However, there were muscles for which those variations of ±1 cm made no sense from a physiological point of view and a smaller variation was analysed [20]. For each of the 19 muscles six simulations were carried out for each attachment point shifted in the three anatomical directions (antero-posterior (AP), cranio-caudal (CC) and latero-medial (LM)) leading to 18 simulations per attachment point. This process resulted in 36 trials per muscle considering the origin and insertion points. Positive shifts point, respectively, in anterior, cranial and lateral directions (Fig 3). The position of attachment points of the 3 actuators of the gluteus (medius, minimus and maximus) and the adductor magnus were modified at the same time in each muscle. Optimal fibre length and tendon slack length were linearly scaled in the same way used in [18,20]. Positions of the auxiliary points (pseudo-origin, pseudo-insertion and via-points) were scaled as well in order to keep the proportions between the different parts of muscles.

For each of those 36 trials, a static optimization problem was solved (Eq (3)) and HJFs were calculated (Eq (5)). These results were compared with those achieved for the baseline model, obtained by means of the generic scaling process implemented in OpenSim.

### 2.4. Methodology to analyse the results

The results obtained in the simulations were analysed from a local and a global point of view. For the local analysis, the procedure proposed Bosmans et al. [20] was used. These authors analysed the anatomical variability only in a representative instant of the gait cycle (the instant t_nom_, where the muscle force peak is reached in the baseline model). Regarding this approach, a new metric was defined to obtain a measure of the deviation relative to peak force:

$$\Delta F_{{mus}_{i}}^{t_{nom}}=\frac{F_{{mod}_{i}}^{t_{nom}}-F_{{base}_{i}}^{t_{nom}}}{F_{{base}_{i}}^{t_{nom}}}$$ (6)

where $F_{{mod}_{i}}^{t_{nom}}$ is the force exerted by muscle *i* at t_nom_ when that is the only modified muscle (this is general from now on when subscript “*mod*_*i*_” is employed), whereas $F_{{base}_{i}}^{t_{nom}}$ is the force exerted by muscle *i* at t_nom_ in the baseline model. Similarly, the deviation of bone-on-bone force in t_nom_ was evaluated with a similar metric:

$${\Delta HJF}_{i}^{t_{nom}}=\frac{{HJF}_{{mod}_{i}}^{t_{nom}}-{HJF}_{base}^{t_{nom}}}{W}$$ (7)

where $H{JF}_{{mod}_{i}}^{t_{nom}}$ is the bone-on-bone force at the hip joint when muscle *i* is modified and ${HJF}_{base}^{t_{nom}}$ is the corresponding bone-on-bone force in the baseline model, The difference between both variables is normalized with the weight of the subject, *W*.

The influence of the anatomical variability in the muscle forces was analysed in [20] only at the instant t_nom_ to ignore the influence of other parameters like the segmental alignment. However, the modification of attachment points yielded changes in the activity of muscles at other instants of the gait cycle and not only at t_nom_, as noticed in [18,19]. For that reason, an average deviation of muscle forces throughout the gait cycle was evaluated by defining the following metric;

$${\tilde{\Delta F}}_{{mus}_{i}}=\frac{1}{T}\int_{0}^{T}\frac{\left(F_{m{od}_{i}}(t)-F_{{base}_{i}}(t)\right)}{F_{{base}_{i}}^{t_{nom}}}dt$$ (8)

where T is the length of the gait cycle; $F_{{base}_{i}}(t)$ is the force exerted by muscle *i* in the baseline model; $F_{{mod}_{i}}(t)$ is the same force, but corresponding to the modified model when *i* is the only modified muscle and $F_{{base}_{i}}^{t_{nom}}$ is the maximum force exerted by muscle *i* in the baseline model throughout the gait cycle. To consider modifications of muscle force patterns due to another kinematic configuration as segmental alignment on a different time instant, the analysis of ${\tilde{\Delta F}}_{{mus}_{i}}$ was complemented with the analysis of moment arm ***r*** during gait cycle. Additionally, the modifications of muscle attachment points might cause variations in their activation pattern making them to be activated at different instants and during a different time. Due to this fact, this study evaluated variables $t_{{act}_{i}}^{base}$ and $t_{{act}_{i}}^{mod}$, which provided the percentage of gait cycle that muscle *i* was activated in the baseline and modified models, respectively.

The sensitivity analysis of bone-on-bone forces in the right hip was carried out using a similar procedure, by normalizing the differences with the weight of the subject through:

$$\tilde{{\Delta HJF}_{i}}=\frac{1}{T}\int_{0}^{T}(\frac{{HJF}_{{mod}_{i}}-{HJF}_{base}}{W})dt$$ (9)

where *HJF*_*base*_ and $H{JF}_{{mod}_{i}}$ are hip bone-on-bone forces in the baseline model and in the modified model when *i* is the modified muscle, respectively.

Regarding the global analysis, a global index was defined to quantify the effect of the modified muscle, *i*, on the force predicted for all the unaltered muscles, *j*. Analogously to the local analysis, two metrics were defined. First, considering only what happens at t_nom_:

$$\Delta GF_{{mus}_{i}}^{t_{nom}}=\frac{\sum_{j\neq i}\left|F_{{mod}_{j}}^{t_{nom}}-F_{{base}_{j}}^{t_{nom}}\right|}{\sum_{j\neq i}F_{{base}_{j}}^{t_{nom}}}$$ (10)

Second, considering the average behaviour throughout the gait cycle:

$${\tilde{\Delta GF}}_{{mus}_{i}}=\frac{1}{T}\frac{\sum_{j\neq i}\int_{0}^{T}|F_{{mod}_{j}}(t)-F_{{base}_{j}}(t)|dt}{\sum_{j\neq i}F_{{base}_{j}}^{t_{nom}}}$$ (11)

### 2.5 Subject-specific model

In addition to the previous general sensitivity analysis and to illustrate it with an example, a particular case of anatomical variation was studied, corresponding to the specific anthropometry of a certain individual. Data available from MRI were taken to define the subject-specific model. In particular, the attachment points of the gluteus medius, gluteus minimus, gluteus maximus and tensor fasciae latae, were modified from the baseline model using MRI data. However, these data were not detailed enough to determine the anatomical variability of the origin and insertion points of the rest of muscles. Thus, the missing information was taken from the baseline model. The muscles scaled from the MRI data and the increment in their positions referred to the baseline model are shown in Table 3. Muscle forces and bone-on-bone forces obtained with the subject-specific model were compared to those obtained with the baseline model.

**Table 3:** Modifications on muscles based on MRI data. Increments in the position of the attachment point locations referred to the baseline model.

Muscles		Gluteus Medius			Gluteus Minimus			Gluteus Maximus			Tensor fasciae latae
		Ant.	Mid.	Post.	Ant.	Mid.	Post.	Ant.	Mid.	Post.	
**Δd Origin** **(mm)**	**AP**	3	-3	-3	3	0	3	-7	-31	-29	6
	**CC**	22	8	41	24	23	22	-10	3	12	-20
	**LM**	-33	2	5	7	7	13	-24	-23	10	3
**Δd Insertion** **(mm)**	**AP**	4	-4	-8	5	3	-5	11	-3	-49	1
	**CC**	5	-12	-23	4	4	3	-5	10	18	4
	**LM**	3	-2	4	-5	-3	-5	-25	-14	-25	-7

The locations of the origin points are relative to the pelvis; the insertion positions are relative to the femur in case of the gluteus. The location of the tensor fasciae latae insertion is relative to the tibia.

## 3. Results

This section shows the results obtained from the sensitivity analysis and the comparison between the two procedures employed to evaluate results. First, results from a local analysis of the muscle forces are shown to analyse how a modification of the attachment point of one muscle affects this own muscle. Second, a global analysis was carried out to analyse how changes in the location of attachment points of one muscle modifies the behaviour of the rest of muscles. Third, the influence of the location of muscle attachment points in the HJFs is shown. Finally, results of the subject specific model are provided.

### 3.1. Local analysis of muscle forces

**Fig 4:**
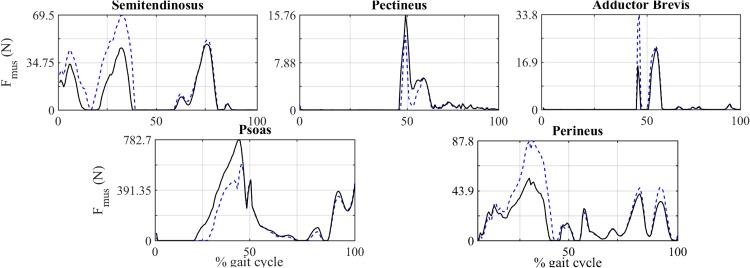
Comparison of the force exerted by five representative muscles throughout the gait cycle. Solid line: baseline model. Dashed line: modified model.

A comparison between muscle forces of some modified models, $F_{{mod}_{i}}$, and the baseline model is shown in Fig 4 and Table 4. These examples show that muscle forces changed significantly when attachment points were modified within the ±1 cm range [20]. A summary of the absolute maximums of ${\tilde{\Delta F}}_{mus}$ due to the modification of each muscle (in origin and insertion) is given in Table 5, as well as the direction for which those maximums are reached. All the maximums were reached at the limits of the interval: |Δ*d*| = 1 cm. The values of $\Delta F_{mus}^{t_{nom}}$ (Eq (6)) evaluated in the same cases are also included.

**Table 4:** Effects on activation pattern.

Muscle	$t_{act}^{base}$ (%)	$t_{act}^{mod}$(%)	${\tilde{\Delta F}}_{mus}$ (%)	${\Delta F}_{mus}^{t_{nom}}$ (%)
Semitendinosus	57.75	67.13	14.21	6.06
Pectineus	53.17	53.17	-3.02	-21.36
Adductor brevis	22.90	22.90	3.18	4.26
Perineus	90.28	94.44	12.04	58.96
Psoas	85.13	81.76	-5.40	-40.12

Percentage of time that the selected muscles (Fig 4) were activated throughout the gait cycle.

**Table 5:** Differences between ${\tilde{\Delta F}}_{\text{mus}}$ and $\Delta F_{\text{mus}}^{t_{nom}}$.

Muscle		Origin			Insertion		
		${\tilde{\Delta F}}_{mus}$ (%)	${\Delta F}_{mus}^{t_{nom}}$ (%)	*Dir*.	${\tilde{\Delta F}}_{mus}$ (%)	${\Delta F}_{mus}^{t_{nom}}$ (%)	*Dir*.
**Gluteus medius**	**Ant**.	-2.99	-9.28	AP	3.48	11.83	AP
	**Mid**.	-2.74	-44.44	AP	1.07	10.18	LM
	**Post**.	10.59	23.31	AP	-1.71	-2.65	CC
**Gluteus minimus**	**Ant**.	-1.23	10.02	LM	-2.68	-5.54	AP
	**Mid**.	-1.88	5.62	LM	-3.09	-4.26	AP
	**Post**.	-3.35	-11.47	AP	4.25	21.96	CC
**Gluteus maximus**	**Ant**.	2.24	7.94	LM	2.92	7.83	LM
	**Mid**.	2.96	10.65	LM	-0.42	1.41	LM
	**Post**.	0.56	0.77	LM	-0.52	-9.14	AP
**Adductor longus**		-1.36	-11.59	AP	-0.42	-4.47	AP
**Adductor brevis**		3.18	4.26	AP	2.07	14.45	AP
**Adductor magnus**	**Ant**.	2.52	-2.84	AP	2.63	11.49	AP
	**Mid**.	2.64	30.04	AP	-0.96	-24.17	AP
	**Post**.	3.83	31.92	LM	0.55	4.89	LM
**Tensor fasciae latae**		-3.06	-26.37	LM	-2.31	-19.67	AP
**Pectineus**		-3.02	-21.36	AP	1.18	16.20	LM
**Iliacus**		2.63	11.72	LM	-4.53	-58.02	LM
**Psoas**		-5.40	-40.12	LM	-4.67	-54.70	LM
**Quadriceps femoris**		0.49	-3.02	AP	0.63	-8.85	CC
**Gemelus**		-2.33	3.13	AP	4.47	-27.62	CC
**Perineus**		4.91	10.91	CC	12.04	58.96	CC
**Rectus femoris**		1.06	0.71	LM	2.29	18.34	AP
**Semimembranosus**		5.39	-0.47	LM	-2.60	-16.18	AP
**Semitendinosus**		14.21	6.06	LM	8.76	27.83	AP
**Biceps femoris long head**		8.77	4.54	LM	4.79	19.98	AP
**Sartorius**		-3.57	-25.16	LM	-2.28	-4.63	AP
**Gracilis**		3.19	24.10	AP	3.73	4.56	AP

Summary of the absolute maximum average deviation of muscle forces when the origin and insertion point of each muscle was modified. *Dir* corresponds to the direction for which that maximum occurred.

A threshold was defined to label the changes in estimated muscle forces as significant or non-significant. The chosen threshold was 20% of the maximum variation for each measure. Therefore, changes of muscle forces at t_nom_ within the range [-10%, 10%] or changes of average deviations within the range [-2%, 3%] were not considered significant.

These changes were caused by modifications of the activation pattern or in the peak force of each muscle. An analysis of the moment arms evolution was carried out to determine if the changes in the results were caused only by modifications of the insertion point locations or, additionally, by particular kinematic configurations.

**Fig 5:**
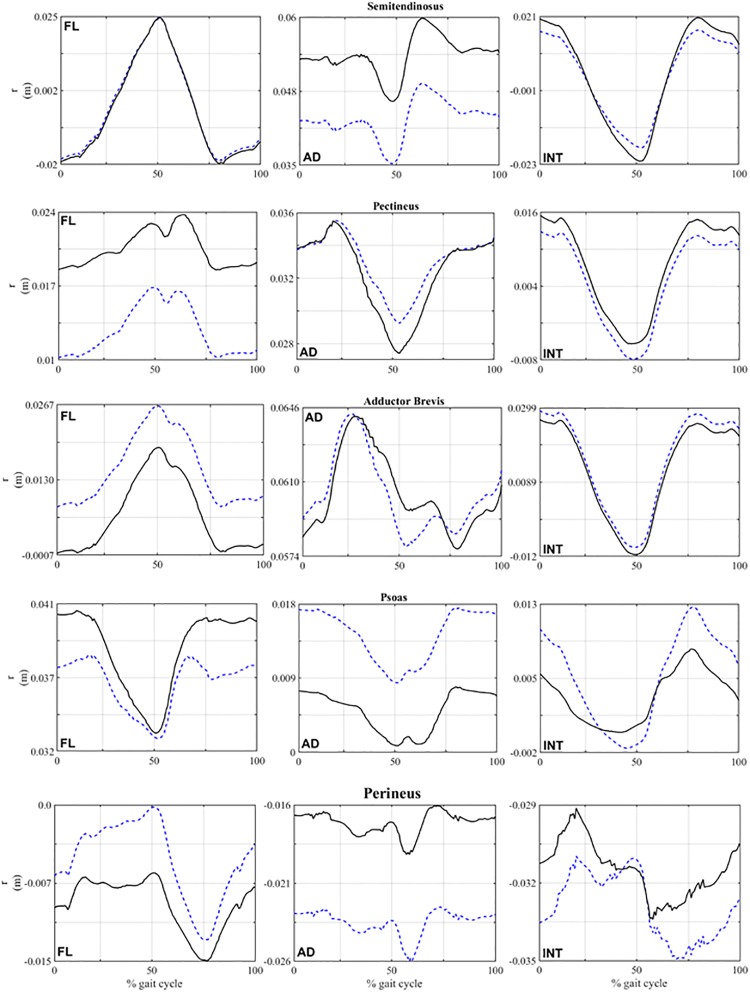
Comparison of the moment arm components of five representative muscles throughout the gait cycle. Solid line: baseline model. Dashed line: modified model.

The cases shown in Fig 4 are a modification of pectineus origin -1 cm and psoas origin -1 cm in AP and LM direction respectively; a modification of adductor brevis origin +1 cm in AP direction, a shift of semitendinosus origin +1 cm in LM direction and, finally, a modification of perineus insertion +1 cm in CC direction. These muscles were chosen because they exhibited different characteristic behaviours. The values of $t_{{act}_{i}}^{base}$ and $t_{{act}_{i}}^{mod}$ of those muscles shown in Fig 4 are compared in Table 4. Temporal evolutions of the projection of the moment arms in the anatomical directions are shown in Fig 5 for those four muscles. The results of the rest of muscles are included in the S2 Table.

**Fig 6:**
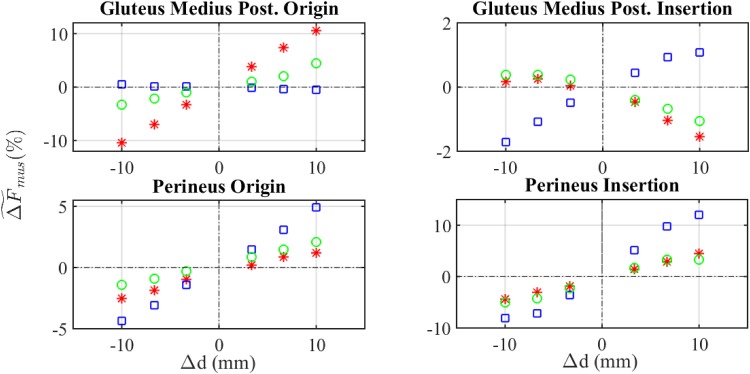
Sensitivity analysis of muscles forces using ${\tilde{\Delta F}}_{\text{mus}}$. Asterisk: shift in AP direction. Square: shift in CC direction. Circle: shift in LM direction.

**Fig 7:**
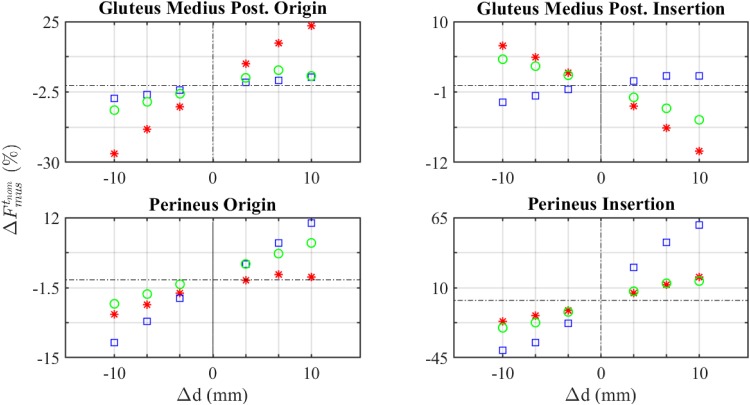
Sensitivity analysis of muscles forces using ${\tilde{\Delta F}}_{\text{mus}}$. Asterisk: shift in AP direction. Square: shift in CC direction. Circle: shift in LM direction.

Fig 6 shows the values of ${\tilde{\Delta F}}_{mus}$ (see Eq (7)) in four representative modifications corresponding to those muscles exhibiting the most significant changes. It can be observed that some modifications in the location of attachment points have no influence on the value of ${\tilde{\Delta F}}_{mus}$, as for example the modification of the origin of gluteus medius posterior in cranio-caudal direction. Other muscles showed a linear variation of ${\tilde{\Delta F}}_{mus}$ with the shift of attachment points, as the perineus, for example. Finally, there was a third group of muscles whose variation of ${\tilde{\Delta F}}_{mus}$ showed a non-linear behaviour, as the gluteus medius posterior. A similar behaviour was observed for ${\Delta F}_{mus}^{t_{nom}}$ (Fig 7). The results of the rest of muscles are given in the S1 Fig.

### 3.2 Global analysis of muscle forces

Table 6 provides the values of the global metrics to quantify the effect of the variation of one muscle on the force exerted by the rest of muscles. Table 7 gives, for two of the muscles with the highest values of the global metrics (Gluteus medius and iliacus), a local analysis of their influence on the rest of muscles. In this particular case, the modified muscles are, respectively, the gluteus medius and iliacus, but the subscript *i* in Eqs (6) and (7) corresponds to the muscles of the rows of Table 7.

**Table 6:** Effect of modifying a muscle on the rest of unperturbed muscles.

Muscle	Origin			Insertion		
	${\tilde{\Delta GF}}_{mus}$ (%)	${\Delta GF}_{mus}^{t_{nom}}$ (%)	*Dir*.	${\tilde{\Delta GF}}_{mus}$ (%)	${\Delta GF}_{mus}^{t_{nom}}$ (%)	*Dir*.
**Gluteus medius**	0.88	7.97	AP	2.04	7.11	AP
**Gluteus minimus**	0.55	4.51	AP	0.54	2.46	AP
**Gluteus maximus**	0.36	0.56	LM	0.32	0.61	LM
**Adductor longus**	0.17	0.17	AP	0.04	0.15	AP
**Adductor brevis**	0.03	0.28	AP	0.02	0.29	AP
**Adductor magnus**	0.07	0.21	AP	0.03	0.34	AP
**Tensor fasciae latae**	0.22	0.67	LM	0.15	0.54	AP
**Pectineus**	0.02	0.03	AP	0.01	0.04	LM
**Iliacus**	3.58	12.56	LM	1.62	13.68	LM
**Psoas**	1.86	9.79	LM	1.77	13.38	LM
**Quadriceps femoris**	0.03	0.07	CC	0.07	0.04	AP
**Gemelus**	0.01	0.01	AP	0.01	0.02	CC
**Perineus**	0.08	0.25	CC	0.35	0.43	CC
**Rectus femoris**	0.46	3.08	LM	0.31	1.60	AP
**Semimembranosus**	0.87	2.52	LM	0.39	2.23	AP
**Semitendinosus**	0.20	0.35	LM	0.14	0.53	AP
**Biceps femoris long head**	0.55	0.66	LM	0.41	1.77	AP
**Sartorius**	0.08	0.44	LM	0.05	0.22	AP
**Gracilis**	0.02	0.11	AP	0.03	0.12	AP

Summary of the values of the global metrics when the origin and insertion point of each muscle was modified. Only the direction for which ${\tilde{\Delta GF}}_{mus}$ was maximum was selected and specified in the column *Dir*. Cells shading (red for ${\tilde{\Delta GF}}_{mus}$ and blue for ${\Delta GF}_{mus}^{t_{nom}}$) is directly proportional to the value of the metrics.

**Table 7:** Effect of modifying gluteus medius and iliacus on the rest of actuators.

Muscle		Gluteus Medius		Iliacus	
		${\tilde{\Delta F}}_{mus}$ (%)	${\Delta F}_{mus}^{t_{nom}}$ (%)	${\tilde{\Delta F}}_{mus}$ (%)	${\Delta F}_{mus}^{t_{nom}}$ (%)
**Gluteus medius**	**Ant**.	3.48	11.83	-3.27	-10.75
	**Mid**.	-0.01	-11.99	0.44	-13.21
	**Post**.	-1.54	-10.26	-3.31	-0.20
**Gluteus minimus**	**Ant**.	-1.75	12.27	11.56	9.06
	**Mid**.	2.68	2.15	14.00	17.99
	**Post**.	-3.69	-17.36	15.29	37.67
**Gluteus maximus**	**Ant**.	0.47	8.08	4.86	13.07
	**Mid**.	1.79	10.56	-0.14	-6.85
	**Post**.	0.56	24.22	-0.35	-57.91
**Adductor longus**		1.23	2.05	2.68	6.75
**Adductor brevis**		0.89	0.76	6.61	16.26
**Adductor magnus**	**Ant**.	0.32	0.48	5.89	12.36
	**Mid**.	0.51	1.67	8.60	-51.23
	**Post**.	3.83	30.01	7.02	54.23
**Tensor fasciae latae**		-3.63	4.28	15.01	16.33
**Pectineus**		0.75	1.03	17.76	117.23
**Iliacus**		-2.09	-8.72	-2.19	-11.84
**Psoas**		-2.29	-13.87	0.81	-1.94
**Quadriceps femoris**		0.14	-0.04	-0.56	-14.43
**Gemelus**		-0.13	-0.22	25.81	74.31
**Perineus**		-4.76	-3.98	-1.19	2.54
**Rectus femoris**		-0.27	-1.96	2.19	14.96
**Semimembranosus**		5.28	0.36	1.98	-20.08
**Semitendinosus**		12.15	0.69	17.66	36.54
**Biceps femoris long head**		4.00	1.01	0.95	0.99
**Sartorius**		-2.58	4.01	18.45	62.72
**Gracilis**		0.58	-1.13	20.38	80.03

Local metrics values of all muscles (rows) when gluteus medius (insertion shifted +1 cm in AP direction) or iliacus (origin shifted -1cm in LM direction) was modified.

### 3.3 Analysis of HJF

**Fig 8:**
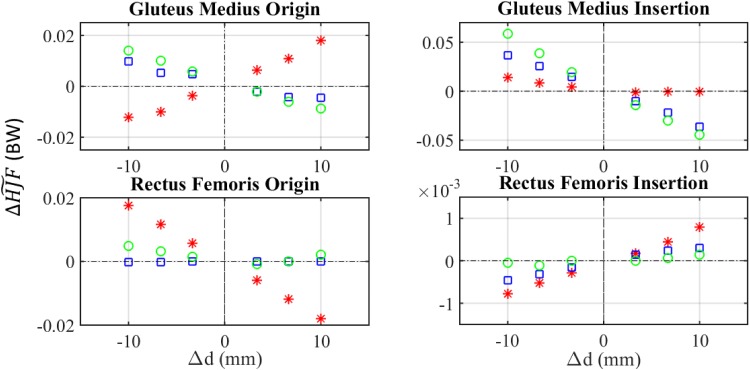
Sensitivity analysis of HJF. Asterisk: shift in AP direction. Square: shift in CC direction. Circle: shift in LM direction.

**Fig 9:**
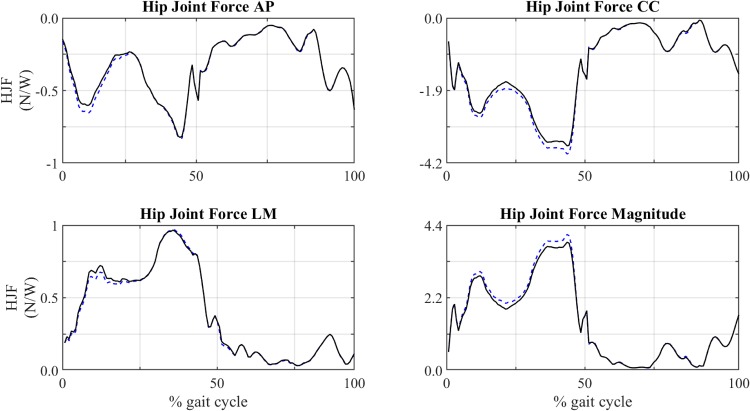
Comparison of the HJF of the baseline and modified model. Evolution of forces exerted throughout the gait cycle when the insertion of gluteus medius was shifted -1 cm in LM direction. Solid line: baseline model. Dashed line: modified model.

The variation of $\tilde{\Delta HJF}$ is represented in Fig 8 for four muscles. ${\Delta HJF}^{t_{nom}}$ is not shown since its behaviour is quite similar to $\tilde{\Delta HJF}$, as it occurred with the analysis of muscle forces. The muscles whose modification is analysed in that Fig 8 are gluteus medius and rectus femoris, because they had the most significant influence on the behaviour of HJF, as will be discussed below. Table 8 shows a summary of the absolute maximums of $\tilde{\Delta HJF}$ due to the modification of attachment points of each muscle as well as the direction for which the corresponding maximum occurred. All the maximums were reached at the limits of the interval: |Δ*d*| = 1 cm. The values of ${\Delta HJF}^{t_{nom}}$ (see Eq (7)) evaluated in the same cases are also included. Fig 9 shows the temporal evolution throughout the gait cycle of the components and magnitude of HJF when gluteus medius insertion was shifted -1 cm in LM direction. The influence of other modifications is included in S2 Fig and S3 Table.

**Table 8:** Differences between $\tilde{\Delta \text{HJF}}$ and ${\Delta \text{HJF}}^{t_{nom}}$.

Muscle	Origin			Insertion		
	$\tilde{\Delta HJF}$ (%BW)	${\Delta HJF}^{t_{nom}}$ (%BW)	*Dir*.	$\tilde{\Delta HJF}$ (%BW)	${\Delta HJF}^{t_{nom}}$ (%BW)	*Dir*.
**Gluteus medius**	1.79	8.0	AP	5.90	23.53	LM
**Gluteus minimus**	-0.61	-5.83	AP	-1.08	-5.20	CC
**Gluteus maximus**	0.69	0.0	AP	-0.51	0.0	AP
**Adductor longus**	-0.20	0.0	AP	-0.02	0.0	AP
**Adductor brevis**	-0.04	0.0	AP	-0.03	0.0	AP
**Adductor magnum**	-0.14	0.0	AP	-0.04	0.0	AP
**Tensor fasciae latae**	-0.45	-2.67	LM	-0.36	0.67	AP
**Pectineus**	-0.02	0.0	LM	-0.01	0.0	AP
**Iliacus**	-8.74	-4.35	LM	-1.94	-14.97	AP
**Psoas**	-3.19	21.03	CC	2.26	-18.22	CC
**Quadriceps femoris**	0.20	0.0	CC	0.17	0.0	CC
**Gemelus**	-0.02	0.0	CC	-0.05	0.0	CC
**Perineus**	0.16	0.0	AP	-0.72	-0.03	CC
**Rectus femoris**	1.79	1.37	AP	0.08	0.0	AP
**Semimembranosus**	1.90	16.45	LM	-0.97	0.0	AP
**Semitendinous**	0.36	0.0	LM	-0.37	0.0	AP
**Biceps femoris long head**	1.33	0.0	LM	1.05	0.0	AP
**Sartorius**	-0.23	-1.22	AP	-0.10	0.13	AP
**Gracilis**	-0.03	0.0	AP	-0.04	0.0	AP

Summary of the absolute maximum of average deviation of resultant hip bone-on bone force when the origin and insertion points of each muscle were modified. *Dir* corresponds to the direction for which that maximum occurred.

### 3.4 Subject-specific model

**Fig 10:**
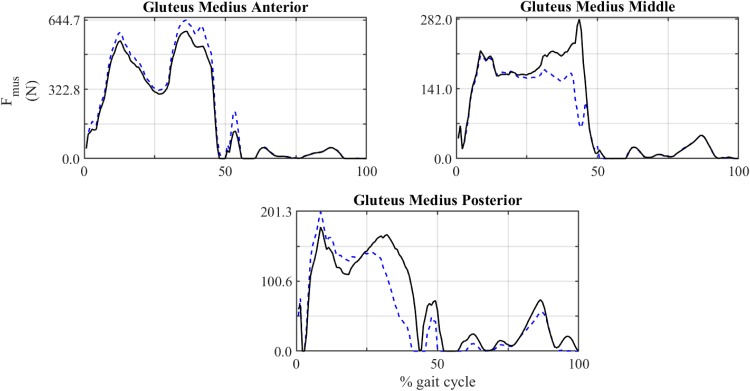
Comparison of the muscle force exerted by gluteus medius in baseline and subject-specific model. Solid line: baseline model. Dashed line: subject-specific model.

**Fig 11:**
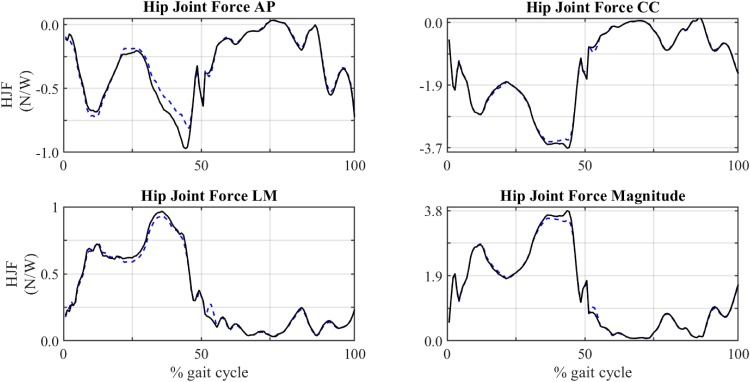
Comparison of HJF obtained in baseline and subject-specific model. Solid line: baseline model. Dashed line: subject-specific model.

Figs 10 and 11 and Table 9 show the results of the subject-specific model. The comparison of the force exerted by gluteus medius during the gait cycle between this model and the baseline model is shown in Fig 10. A comparison of the temporal evolution of the components and magnitude of HJF between both models is shown in Fig 11. Table 9 provides the relative difference between the muscle forces obtained with both models: at t_nom_ ($\Delta F_{mus}^{t_{nom}}$) and averaged throughout the gait cycle (${\tilde{\Delta F}}_{mus}$).

**Table 9:** Effect of MRI data.

Muscle		$F_{base}^{t_{nom}}$ (N)	$F_{mod}^{t_{nom}}$ (N)	${\tilde{\Delta F}}_{mus}$ (%)	${\Delta F}_{mus}^{t_{nom}}$ (%)
**Gluteus medius**	**Ant**.	591.51	644.66	3.22	8.98
	**Mid**.	282	69.01	-4.28	-75.53
	**Post**.	179.12	201.34	-6.67	12.41
**Gluteus minimus**	**Ant**.	123.11	202.93	7.41	64.80
	**Mid**.	98.43	139.05	8.40	41.26
	**Post**.	82.59	117.49	10.14	42.26
**Gluteus maximus**	**Ant**.	81.15	43.29	-12.26	-46.65
	**Mid**.	140.2	113.04	-4.86	-19.38
	**Post**.	121.1	83.38	-0.99	-31.15
**Tensor fasciae latae**		92.36	132.72	3.81	43.70

Forces exerted by those muscles whose attachments were located through MRI data in the subject-specific model. The differences were evaluated using Eqs (6) and (8).

## 4. Discussion

This work proposes a new method to study the sensitivity of musculoskeletal models to variations of the location of muscle attachment points which can complement and improve previously developed methods. Bosmans et al [20] developed a useful method to quantify that sensitivity consisting in the comparison of the peak force exerted by the modified muscle. However, this method cannot quantify changes in other instants of the gait cycle. Valente [18] and Carbone [19] did use metrics to consider the whole cycle. However, those procedures could be affected by particular kinematic configurations. The goal of this work was to propose a method to evaluate this sensitivity in a more complete way. The method developed considers not only the peak force exerted by a muscle but it also quantifies the changes throughout the whole gait cycle. Comparing the average deviation of forces throughout the gait cycle allows evaluating changes in the muscular activation pattern. Another goal of this work was to extend the sensitivity analysis to the study of the variations in the HJF, considering not only the instant where the muscle reaches the peak force but also the whole gait cycle.

### 4.1. Local analysis of muscle forces

Regarding the new approach proposed in this work, different behaviours could be observed in the local analysis. The case of semitendinosus, analysed in section 3.1, is a representative example of the benefits of the new method. Analysing Fig 4 and Table 4, it can be observed that the peak force (around the 75% if the gait cycle) was hardly 6% higher when attachment points were modified, but the evolution was very different and the average deviation was 14.21%, mainly due to the activation between the 12% and the 22% of the gait cycle. For this particular case, the differences between $t_{{act}_{i}}^{base}$ (57.75%) and $t_{{act}_{i}}^{mod}$ (67.13%) reflected that modifications of the geometry of muscles may also affect the activation pattern in a significant manner. The functions of this muscle are the extension and adduction of the hip. The analysis of the moment arm (Fig 5) showed that no significant variations occurred in the flexion component. However, the adduction component of the moment arm showed a shift of -1 cm. This variation may be responsible for the change in the activation pattern. Additional examples of this behaviour occurred when the location of the origin point was modified in the case of semimembranosus and biceps femoris longus (Table 5 and S1 Table). In these cases, a simple analysis of the variations in the peak muscle force would have failed to detect an influence of the geometry on muscle forces, as the present method does.

A second type of behaviour could be observed in Fig 4 and Table 4 by analysing the results of pectineus. In this case ${\Delta F}_{mus}^{t_{nom}}$ is -21.36% whereas the average deviation ${\tilde{\Delta F}}_{mus}$ is only -3.02%. These results showed that modifying the geometry just led to a decrease in the level of activation close to the instant where the muscle force peak is reached, as shown in [20], but no significant change occurred in the time of activation, as can be observed from the comparison of $t_{{act}_{i}}^{base}$(53.17%) and $t_{{act}_{i}}^{mod}$ (53.17%). The functions of pectineus are the flexion and adduction of the hip. The main modification in the moment arm was in the flexion component around 8 mm (Fig 5). However, this variation in the moment arm had not significant influence in the time of activation. Table 5 shows additional examples of this behaviour: iliacus, gracilis and gluteus minimus.

A third type of behaviour was observed, viz. the case in which ${\Delta F}_{mus}^{t_{nom}}$ and ${\tilde{\Delta F}}_{mus}$ showed similar values as occurred with modifications in the origin of adductor brevis (see Fig 4 and Table 4). Changes of 1 cm occurred in the temporal evolution of the flexion component of moment arm (Fig 5). However, no significant changes were observed in the temporal evolution of muscle forces which excludes the influence of particular kinematic configurations on the activation pattern. Another example of this behaviour is shown in the modifications of the origin of gluteus maximus posterior (Table 5 and S1 Table).

Finally, a fourth type of behaviour was shown analysing the behaviour of the perineus when its insertion point was modified. Regarding the values of the metrics shown in Table 4, both ${\Delta F}_{mus}^{t_{nom}}$ and ${\tilde{\Delta F}}_{mus}$ had a high value whereas the time of activation showed a slight modification. This change in the activation pattern could be explained by analysing the temporal evolution of its moment arms. The functions of the perineus is abduction and external rotation. Small changes in these components of its moment arms during the first and the last phases of the gait cycle are correlated with changes in the activation pattern. On the other hand, in the range of 40–55% of the gait cycle, no significant changes occurred. A similar behaviour is shown in psoas (Fig 4 and Table 4). In this case ${\Delta F}_{mus}^{t_{nom}}$ is -40.12% while ${\tilde{\Delta F}}_{mus}$ is -5.40%, slightly smaller than the value for perineus. Comparing the time of activation ($t_{{act}_{i}}^{base}=85.13\%$ vs. $t_{{act}_{i}}^{mod}=81.76\%$) a moderate change in the activation pattern is observed, which it is reflected in the average deviation ${\tilde{\Delta F}}_{mus}$. This variation could be explained by analysing the temporal evolution of the moment arm. The functions of psoas are flexion and internal rotation. The modifications in the activation pattern occurred around 20–30% of the gait cycle. In this interval, the flexion and internal rotation components of moment arm underwent variations of 4 and 3 mm, respectively.

The type of behaviour of each muscle was summarized in S2 Table.

Numerical values of both metrics shown in Table 5 were within very different ranges. ${\tilde{\Delta F}}_{mus}$ was between -5.40 and 14.21% whereas ${\Delta F}_{mus}^{t_{nom}}$ was between -58.02 and 58.96%. Therefore, the behaviour of one muscle when the insertion point was modified could be understood by means of the two metrics. For example, in the case of gluteus medius posterior, the value of ${\tilde{\Delta F}}_{mus}=10.59$ meant a significant change in the activation pattern and a value of ${\Delta F}_{mus}^{t_{nom}}=23.21$ suggested a moderate change in the peak force.

Regarding local metrics, ${\tilde{\Delta F}}_{{mus}_{i}}$ and ${\Delta F}_{mus}^{t_{nom}}$, they showed a certain linearity with the shift of attachment points, Δd, in most muscles (Figs 6 and 7). However, some nonlinearities could be observed for the insertion of gluteus medius posterior, no matter the metric employed. Additional examples of nonlinearities were found in gluteus maximus, psoas and semimembranosus (S1 Fig). These results are in agreement with Carbone et al. [19], who reported a low linear correlation coefficient in these cases.

### 4.2. Global analysis of the muscle forces

Section 3.2 analysed how the modification of the attachment points of one muscle affected the forces exerted by the rest of muscles (Table 6) by means of two global metrics. The muscles with the highest values of these metrics were gluteus medius, iliacus and psoas, which was in agreement with [20]. The gluteus medius plays an important role in abduction and rotation movements, as it was established in [18–20]. Besides, this muscle also plays a minor role in hip extension according to [27]. As a consequence, changes in this muscle affect very importantly the other muscles involved in abduction (gluteus minimus, perineus, tensor fasciae latae and sartorius), rotation (gluteus minimus, tensor fasciae latae, perineus, psoas and iliacus) and extension (gluteus minimus, semitendinosus, semimembranosus and biceps femoris long head). The values of ${\tilde{\Delta F}}_{mus}$ and ${\Delta F}_{mus}^{t_{nom}}$ shown in Table 7, reinforced that idea since the referred muscles exhibit significant values of those metrics. However, deviations were irrelevant for the rest of muscles.

As previously commented, another muscle with a high value of the average deviation was the iliacus, which plays an important role in hip flexion and internal rotation. Table 7 shows that variations in this muscle affect a significant number of muscles in noteworthy values of ${\tilde{\Delta F}}_{mus}$ and ${\Delta F}_{mus}^{t_{nom}}$, which may be due to the high number of muscles involved in hip flexion (sartorius, gracilis, pectineus) and rotation (gluteus medius and minimus and tensor faciae latae).

These results highlighted that these two muscles reflect different behaviours. Variations in the gluteus medius cause significant values of the metrics only in a small number of muscles (semitendinosus and semimembranosus), whilst modifications of iliacus affect a large number of muscles and in a more significant way.

### 4.3 Analysis of HJF

In addition to the sensitivity analysis of muscle forces, similar to those made in [19–20], a sensitivity analysis of HJF was also performed, as in [18]. This analysis showed again a certain linearity of HJF average deviation with the shift of attachment points (Fig 8), but in this case the sensitivity was lower than that of muscle forces (Table 8), the reason being that HJF includes the effect of all muscle forces. Thus, the changed and unchanged muscles modified their forces to compensate the net moment and this fact mitigated the consequences of modifying attachment points. Table 8 also reflected that the muscles which caused the largest variation in HJF were those whose force peak was closest to the instant of maximum HJF magnitude (44% of the gait cycle) and their effect could be evaluated as in [20], just by analysing the force peak, for example, the gluteus medius, the iliacus or the psoas (see supplementary material S2 Table for more information). Regarding the gluteus medius, a shift in its insertion produced a $\tilde{\Delta HJF}=5.90\%$ and ${\Delta HJF}^{t_{nom}}=23.93\%$. Meanwhile, the effect of those muscles whose peak occurred in a different instant was better captured if HJF was compared throughout the whole gait cycle as was shown in Table 8 in case of gluteus maximus and rectus femoris. $\tilde{\Delta HJF}$ was used to compare the results obtained here which those previously shown in the literature [21]. Valente et al. [18] established that gluteus medius anterior, iliacus and psoas were the muscles with the strongest influence on HJF. This is in agreement with the results obtained here in view of Table 8, which shows that those muscles, together with semimembranosus, produced the highest values of $\tilde{\Delta HJF}$.

Fig 9 shows the evolution of components and magnitude of HJF throughout the gait cycle. The HJF magnitude reached a peak of 2864.63 N and 3006.97 N in the baseline and in the modified models, respectively. The CC component is clearly predominant, especially at the peak (44% of the gait cycle), which makes the evolution of the magnitude to be very similar to that component. Due to this fact ${\Delta HJF}^{t_{nom}}$ mainly reflected the variation of the CC component, whereas $\tilde{\Delta HJF}$ reflected the effects of the three components altogether, because their values are more similar during the rest of the gait cycle. Therefore, $\tilde{\Delta HJF}$ describes better than ${\Delta HJF}^{t_{nom}}$ the sensitivity of the model in those muscles whose modification affects importantly the AP and LM components of HJF. Examples of those modifications are both attachments of biceps femoris longus and the origin of iliacus, which produced variations of LM components in the range 1–8.7% of body weight and both attachments of gluteus maximus which produce variations of AP components in the range of 0.5–0.6% of the body weight at instants other than the force peak (see Table 8). The results for the rest of muscles were included in S3 Table.

### 4.4 Subject-specific model

After the sensitivity analysis, section 3.4 studied a subject-specific model with modification of certain attachment points based on MRI data. The results shown in Table 9 and Figs 10 and 11 reinforced the results obtained in the sensitivity analysis. Data taken from MRI confirmed that the analysed muscles (gluteus medius, maximus, minimus and tensor fasciae latae) were longer in this subject than in the baseline model with modifications up to 4 cm in the origin of the posterior part of the gluteus medius posterior and up to 5 cm in the insertion of the posterior part of the gluteus maximum. These modifications produced significant variations in estimated muscle forces and hip bone-on-bone forces. Regarding variations in the peak force, there were decrements up to -75.53% in case of gluteus medius. Regarding the average deviation, gluteus medius and gluteus maximus showed deviations up to -10.14% and -12.26%, respectively. The analysis of HJF evolution throughout the gait cycle (Fig 11) showed deviations up to 35% of body weight in the case of the antero-posterior component.

One limitation of this study was that the data taken from MRI were not detailed enough to determine the anatomical variability of the attachment points of all the muscles involved in hip movement. As another limitation, the analysis was focused on the hip joint; however, several muscles involved in the hip function are bi-articular. Therefore, it would be interesting to carry out the sensitivity analysis considering all the joints related to those muscles.

Since this work uses a computational tool like OpenSim, the limitations of the presented results are conditioned by the limitations of that tool. Nonetheless, it must be clear that the evolution of muscular forces or joint forces obtained here are not to be taken as necessarily true. The only purpose of this work was to check if they change when the attachment points are modified. Therefore, a comparison with a gold-standard measurement was not essential. However, it would be interesting to study if the results obtained in this work are correlated with experimental data, for instance, taken from people with instrumented implants like those available in OrthoLoad database [28].

For example, one of the limitations of OpenSim models is that hip joint centers are excessively close [29,30]. This limitation may have an important effect on the simulations, particularly on the kinematics and the predicted ground reaction forces, which, for instance, could cause abduction rather than adduction in some extreme cases. The latter was not the case in any of the simulations conducted in the present work. Additionally, that limitation could have an effect on the sensitivity analysis performed here. For this reason, the position of the hip joint center is another test variable whose sensitivity should be addressed in future studies, separately or combined with the sensitivity to modifications of the attachment points.

## 5. Conclusions

The present study evaluated the sensitivity of muscle forces and hip bone-on-bone forces estimated through musculoskeletal models to modifications of attachment points. In addition, a subject-specific model was analysed to study how those changes affected the results in a particular case.

Variations of ±1 cm in the position of the attachment points of certain muscles caused variations of up to 14.21% in ${\tilde{\Delta F}}_{{mus}_{i}}$ and 58.96% in ${\Delta F}_{mus}^{t_{nom}}$ in the modified muscle and variations up to 57.23% in ${\tilde{\Delta F}}_{{mus}_{i}}$ and 117.23% in ${\Delta F}_{mus}^{t_{nom}}$ in the rest of muscles. For the estimation of muscular forces, it seems critical to locate accurately those points in a subject-specific musculoskeletal model. Regarding HJF, the analysis showed variations up to 8.7% in $\tilde{\Delta HJF}$ and 23.53% in ${\Delta HJF}^{t_{nom}}$. These variations are not very significant, however, the lateromedial component is highly influenced by the changes in the position of attachment points and this could have important effects, for instance, on the stresses produced in the femoral neck.

Comparing the average deviation of forces throughout the gait cycle seems a good way to complement the comparisons made in previous studies, which were limited to evaluating the force peak, because this last option is unable to detect changes in muscular activation patterns. Evaluating the average deviation could be a good method to analyse the influence of specific kinematic configurations, as segment alignments, in activation patterns.

Modification of the geometry of one muscle caused variations in the forces exerted by the muscle itself and by other muscles. Variations in the modified muscle depended on the distance and direction of the specific modification. Variations in the other muscles depended, besides, on the contribution of the modified muscle in a certain movement. For example, all muscles involved in abduction were affected by a modification of any another muscle responsible for that movement. It has been demonstrated that two muscles with a large value of a global metric can influence the rest of muscles in different ways.

Variations in muscle forces caused, in turn, variations in bone-on-bone forces, which were one order of magnitude smaller than variations in muscle forces. Anyway, these differences can be up to 9% of body weight as occurred, for example, when the origin of the iliacus was shifted -1 cm in LM direction.

The analysis of HJF reflected that CC was the greatest component. However, the study of AP and LM components was interesting because these components experienced changes in the range 0.1–9% of body weight, but at instants other than the force peak.

A biomechanical analysis of a subject-specific musculoskeletal model was carried out, showing significant differences between the baseline model and the customized model: within the range [-12%, 10%] for muscle forces and around 35% of body weight for hip bone-on-bone forces. Regarding the CC component of the HJF, a change of 25% BW was observed around 40–45% of the gait cycle. Since this change was very localized and the range of this HJF component was up to 370% BW, we assumed that this variation was not significant. In the AP component, a change of 35% BW occurred in the same phase of the gait cycle. In this case, the maximum value of this component was up to 100% BW. Therefore, we concluded that this variation was important. These differences reflected the high sensitivity of the model and highlighted the importance of locating accurately muscle attachment points in musculoskeletal models.

## Supporting information

S1 Fig Linearity of muscles forces when the attachment points of muscles were modified from their original position. (DOCX)

S2 Fig Linearity of HJF when the attachment points of muscles were modified from their original position. (DOCX)

S1 Table Temporal evolution of muscle forces for each simulation. Each sheet corresponds to the modifications of one muscle. Each column corresponds to a specific modification.
(XLSX)

S2 Table Classification of the muscles according the four behaviours defined in section 4.1. (DOCX)

S3 Table Temporal evolution of normalized hip joint force. Each sheet corresponds to the modifications of one muscle. Each column corresponds to a specific modification.
(XLSX)
