# Supplementary material for: Influence of musculotendon geometry variability in muscle forces and hip bone-on-bone forces during walking
Source: PLoS One. 2019 Sep 25;14(9):e0222491. doi: 10.1371/journal.pone.0222491 (PMC6760798; doi:10.1371/journal.pone.0222491)
Supplement: S2 Fig — (DOCX) [file pone.0222491.s002.docx]

Figure S2. Linearity of HJF when the attachment points of the muscles were modified from the original position.

Asterisk: Modification in AP direction. Square: Modification in CC direction. Circle: modification in LM direction

Asterisk: Modification in AP direction. Square: Modification in CC direction. Circle: modification in LM direction
