# Supplementary material for: Influence of musculotendon geometry variability in muscle forces and hip bone-on-bone forces during walking
Source: PLoS One. 2019 Sep 25;14(9):e0222491. doi: 10.1371/journal.pone.0222491 (PMC6760798; doi:10.1371/journal.pone.0222491)
Supplement: S2 Table — (DOCX) [file pone.0222491.s004.docx]

| **Muscle** | | **Origin** | | | | **Insertion** | | | |
| --- | --- | --- | --- | --- | --- | --- | --- | --- | --- |
|  |  | **Type of behaviour** | | | | **Type of behaviour** | | | |
|  |  | **1** | **2** | **3** | **4** | **1** | **2** | **3** | **4** |
| **Gluteus**  **medius** | **Ant.** | X |  |  |  |  |  | X |  |
|  | **Mid.** |  |  | X |  |  | X |  |  |
|  | **Post.** |  |  | X |  |  |  |  | X |
| **Gluteus**  **minimus** | **Ant.** |  | X |  |  | X |  |  |  |
|  | **Mid.** |  |  |  | X | X |  |  |  |
|  | **Post.** |  |  | X |  |  |  | X |  |
| **Gluteus**  **maximus** | **Ant.** |  |  |  | X |  |  |  | X |
|  | **Mid.** |  | X |  |  |  |  |  | X |
|  | **Post.** |  |  |  | X |  |  |  | X |
| **Adductor longus** | |  | X |  |  |  | X |  |  |
| **Adductor brevis** | | X |  |  |  |  |  | X |  |
| **Adductor magnus** | **Ant.** |  |  |  | X |  | X |  |  |
|  | **Mid.** |  | X |  |  |  | X |  |  |
|  | **Post.** |  |  | X |  |  |  |  | X |
| **Tensor fasciae latae** | |  |  | X |  |  |  | X |  |
| **Pectineus** | |  |  | X |  |  | X |  |  |
| **Iliacus** | |  | X |  |  |  |  | X |  |
| **Psoas** | |  |  |  | X |  |  |  | X |
| **Quadriceps femoris** | |  |  |  | X |  | X |  |  |
| **Gemelus** | | X |  |  |  |  |  | X |  |
| **Perineus** | |  |  | X |  |  |  | X |  |
| **Rectus femoris** | |  |  |  | X |  | X |  |  |
| **Semimembranosus** | | X |  |  |  |  |  | X |  |
| **Semitendinosus** | | X |  |  |  |  |  | X |  |
| **Biceps femoris long head** | | X |  |  |  |  |  | X |  |
| **Sartorius** | |  |  | X |  | X |  |  |  |
| **Gracilis** | |  |  | X |  | X |  |  |  |

**Table S3.** **Classification of the muscles according to the types of behaviour defined in section 4.1.**

**Behavior type 1:** The value of ${\tilde{\boldsymbol{\Delta F}}}_{\mathbf{mus}}$ is outside the threshold and the value $\boldsymbol{\Delta}\mathbf{F}_{\mathbf{mus}}^{\boldsymbol{t}_{\boldsymbol{nom}}}$ is within the range. **Behavior type 2** The value of ${\tilde{\boldsymbol{\Delta F}}}_{\mathbf{mus}}$ is within the range and the value $\boldsymbol{\Delta}\mathbf{F}_{\mathbf{mus}}^{\boldsymbol{t}_{\boldsymbol{nom}}}$ is outside the threshold. **Behavior type 3:** The values of ${\tilde{\boldsymbol{\Delta F}}}_{\mathbf{mus}}$ and $\boldsymbol{\Delta}\mathbf{F}_{\mathbf{mus}}^{\boldsymbol{t}_{\boldsymbol{nom}}}$ are within the range. **Behavior type 4:** The values of ${\tilde{\boldsymbol{\Delta F}}}_{\mathbf{mus}}$ and $\boldsymbol{\Delta}\mathbf{F}_{\mathbf{mus}}^{\boldsymbol{t}_{\boldsymbol{nom}}}$ are outside the range.
